# Comparative endurance testing of the Biomet Matthews Nail and the Dynamic Compression Screw, in simulated condylar and supracondylar femoral fractures

**DOI:** 10.1186/1475-925X-7-3

**Published:** 2008-01-21

**Authors:** Laurence M O'Connor-Read, Jerome A Davidson, Benjamin M Davies, Michael G Matthews, Paul Smirthwaite

**Affiliations:** 1 Department of Trauma and Orthopaedics, Buckinghamshire Hospitals NHS Trust, High Wycombe, UK; 2 Department of Trauma and Orthopaedics, Milton Keynes Hospital NHS Trust, Milton Keynes, UK; 3 Biomet UK Ltd, Bridgend, UK

## Abstract

**Background:**

The dynamic compression screw is a plate and screws implant used to treat fractures of the distal femur. The Biomet Matthews Nail is a new retrograde intramedullary nail designed as an alternative surgical option to treat these fractures. The objective of this study was to assess the comparative endurance of both devices.

**Method:**

The dynamic compression screw (DCS) and Biomet Matthews Nail (BMN) were implanted into composite femurs, which were subsequently cyclically loaded using a materials testing machine. Simulated fractures were applied to each femur prior to the application of load. Either a Y type fracture or a transverse osteotomy was prepared on each composite femur using a jig to enable consistent positioning of cuts.

**Results:**

The Biomet Matthews Nail demonstrated a greater endurance limit load over the dynamic compression screw in both fracture configurations.

**Conclusion:**

The distal locking screws pass through the Biomet Matthews Nail in a unique "cruciate" orientation. This allows for greater purchase in the bone of the femoral condyle and potentially improves the stability of the fracture fixation. As these fractures are usually in weak osteoporotic bone, the Biomet Matthews Nail represents a favourable surgical option in these patients.

## Background

**Figure 1:**
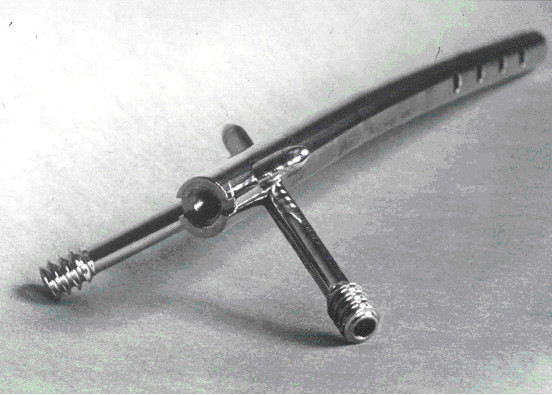
The Retrograde Biomet Matthews Nail.

**Figure 2:**
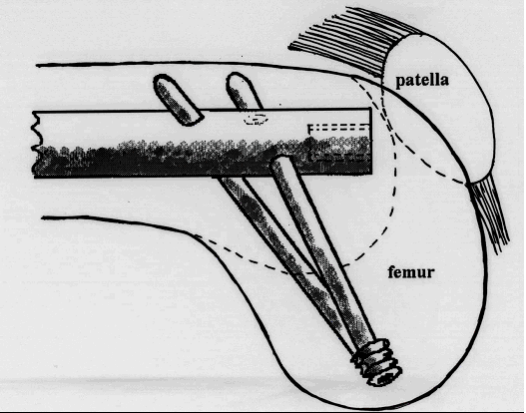
Saggital view of the "cruciate" orientation of the distal locking screws in the femoral condyles.

**Figure 3:**
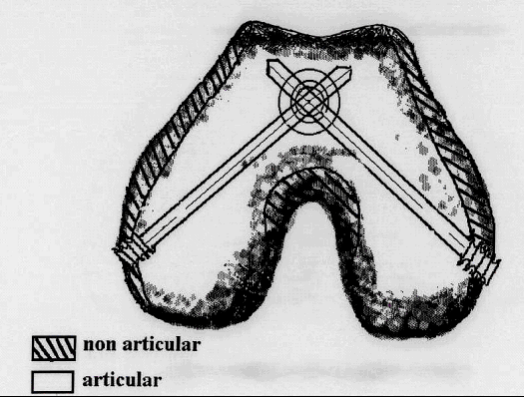
Axial view of the "cruciate" orientation of the distal locking screws in the femoral condyles.

Fractures of the femur at or just above the knee (condylar and supracondylar) comprise 4–7% of all femoral fractures and are considered to represent a challenge to treat [1]. There are principally two groups of patients who typically present with this injury; the elderly female population who present following a minor fall and a smaller but significant group of young patients who are involved in high energy trauma [1]. The elderly patients often have significant weak and osteoporotic bone, which may compromise the fixation of the fracture. The Biomet Matthews Nail (BMN) is a new retrograde nail (passing from the knee to the hip), designed with unique specifications, to treat distal femoral fractures [2]. This original design allows the distal locking screws (Figures 1, 2 &3) to create a "cruciate" arrangement as they pass through the intramedullary nail. By using the anatomical contours of the distal femur, these distal locking screws pass through a greater amount of the bone, within the femoral condyles and gain greater purchase in weak osteoporotic bone. The dynamic compression screw (DCS), which is a plate and screw implant, is an established alternative treatment to the intramedullary nail in these injuries [3-6].

A preliminary report by Henry et al [7] on the use of intramedullary nails with supracondylar femoral fractures suggested an improved stability over the plate and screw system because of a reduced moment arm. Firoozbakhsh et al [8] compared the rigidity of another type of intramedullary nail to the dynamic compression screw and suggested that although the DCS performed better in their tests overall, the rigidity of their intramedullary device was equivalent to plate and screw fixation during clinical modes of loading and therefore could be considered as a mechanically reasonable alternative.

The objective of this study was to assess the comparative mechanical endurance performance of the new Biomet Matthews Nail and the Dynamic Condylar Screw, when dynamically tested in Y fractured femurs and transverse osteotomised composite femurs.

## Methods

The intramedullary nails and dynamic compression screws were implanted into composite femurs which were subsequently cyclically loaded using a materials testing machine. Simulated fractures were applied to each femur prior to the application of the load. Either a Y type osteotomy or a transverse osteotomy was prepared on each composite femur using a jig to enable consistent positioning of cuts.

### Design of the Biomet Matthews Nail

The intramedullary nail is a solid stainless steel rod available in two diameters; 12 and 14 mm and four different lengths; 200, 250, 300 and 350 mm. The nail has a single radius of curvature of 2290 mm. The geometry of the nail permits its use for both left and right femurs (Figure 1).

The distal locking screws pass through the femoral condyles, posterior to the intramedullary line, in a unique "cruciate" orientation to achieve maximum bone purchase (Figure 2 &3).

### Test specimens

• Eight Biomet Matthews Nails (12 and 14 mm diameters). The two sizes differed only in their diameter superior to the distal locking screw holes. Therefore nail performance, under test, in the distal screw region was considered to be equivalent for both sizes.

• Four 95 degree suprcondylar plates (4 off Supraloc CCS Plate)

• Four Lag screws (4 off Hiploc CHSS lag screw 12.5 Diameter)

• Biomet ST-PRO fixation screws (6 mm × 35 mm: 6 mm × 40 mm: 6 mm × 45 mm:)

• Biomet Cancellous bone screws (6.5 mm × 32 mm thread, length = 40 mm)

### Implantation of nails and plates into composite femurs

The nails and dynamic compression screws were implanted into left composite femurs (Sawbones Europe AB, Malmö, Sweden) by MGM using the appropriate sets of instruments. Fixation screws were inserted through the nails, using the two most inferior cross-holes. Fixation screws were secured through each hole of the plates except for the hole immediately superior to the lag screw, which received a cancellous bone screw. Radiographs of the femur were taken following the insertion of each fixation device, to confirm their correct placement.

### Cutting of Y fractures & transverse osteotomies into composite femurs

The composite femoral cuts were determined using the AO classification, one of the most commonly used classifications for distal femoral fractures [9]. The transverse osteotomy represented the 33A type (extra-articular) fracture and the Y shaped osteotomy represented the challenging 33C type (bicondylar intra-articular) fracture. The 33B type (unicondylar) fractures are less commonly seen in clinical practice and have therefore not been used in this study [10].

**Figure 4:**
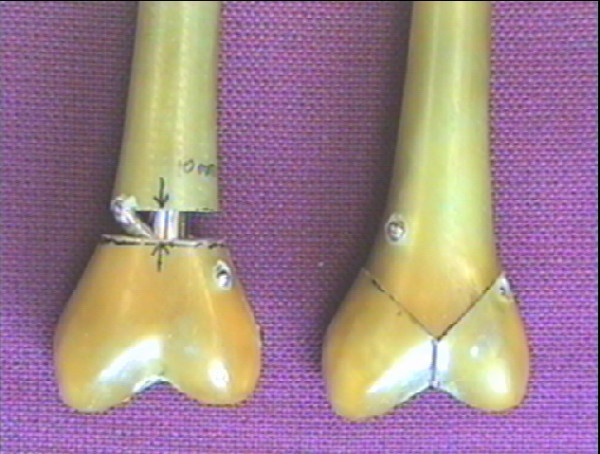
Photograph demonstrating the transverse osteotomy and Y fracture patterns.

All nails, plates and screws were removed from individual femurs prior to any cuts being made. A femur would then be clamped within a custom made bone cutting jig and cuts made using either a Y fracture guide or an osteotomy guide. Figure 4 demonstrates the position of these cuts.

The use of a Y fracture resulted in the condyles being secured only by the fixation device, which therefore experienced considerable loading. Similarly, an osteotomy was used (10 mm wide) to enable the entire load to be transmitted through the nail or the plate. Following cutting all implanted items were reinserted as performed previously.

### Test procedure

**Figure 5:**
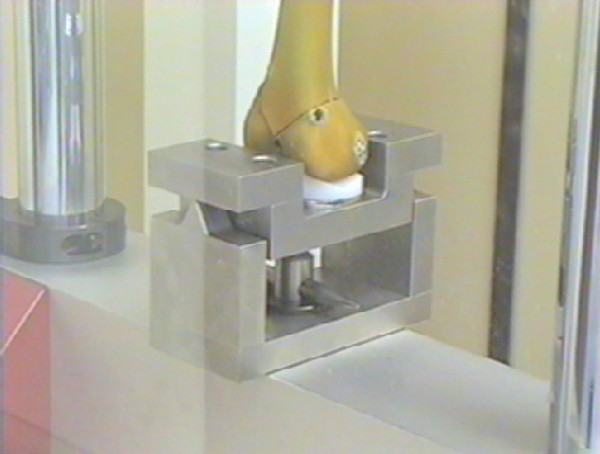
Photograph demonstrating the method of condyle support.

Testing was carried out using a servo-hydraulic materials testing machine (Dartec HC 10, Dartec Ltd.) of 10 kN capacity (National Association Measuring Analysis Systems calibrated). This has a top cross-head mounted actuator, load cell and linear voltage displacement transducer. Femurs were positioned vertically within the test machine with the femoral head uppermost. The femoral condyles were placed on a Denham knee tibial component (Biomet Ltd.), supported in a cradle, which balanced on a knife edge (Figure 5). The bearing surface of this component consisted of a simple trough which therefore provided anterior-posterior constraint but no medio-lateral constraint to the condyles.

**Figure 6:**
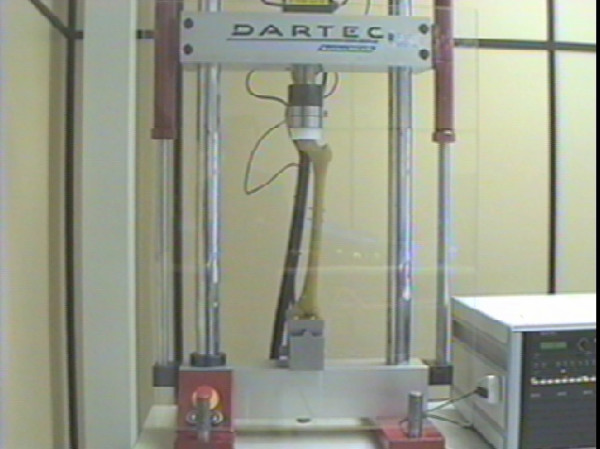
Photograph demonstrating the femur within the test machine.

The knife edge was positioned to allow the tibial component to be free to balance about an anterior-posterior axis, passing over the surface of the tibial bearing. An ultra-high molecular weight polyethylene cylinder, or loading socket, was positioned between the femoral head and the test machine actuator. The lower face of the cylinder was concave to produce a congruent fit with the femoral head. Thus the design of the tibial support ensured that load applied through the femoral head was transmitted equally through both femoral condyles (Figure 6).

### Test conditions and data collection

Sinusoidal loading was applied to each specimen between specified maximum and minimum compressive loads at a frequency of 5 Hz, until 1 million cycles had been executed or specimen failure had occurred (whichever was sooner).

The four groups of test specimens consisted of:

1. BMN in femurs with Y osteotomies

2. BMN in femurs with transverse osteotomies

3. DCS on femurs with Y osteotomies

4. DCS on femurs with transverse osteotomies

Maximum and minimum vertical displacement and vertical displacement amplitude were generally logged twice daily over the course of the 56 hours, or less, as required for each test.

## Results

### Endurance Testing of the Biomet Matthews Nail and Dynamic Compression Screws tested with Y fractured composite femurs (Table 1)

**Table 1:** Endurance testing of the Biomet Matthews Nail and the Dynamic Compression Screw tested with Y fractured composite femurs

	Biomet Matthews Nail with Y fractured femur		Dynamic Compression Screw with Y fractured femur	
Max. & Min. of Load Cycle (kN)	Specimen Number	Cycles Completed	Specimen Number	Cycles Completed

2.0 & 0.2	1	1,000,000	1	1,000,000^#^
2.0 & 0.2	2	315,043	2	1,000,000^#^
3.0 & 0.3	3	22,356^+^		
3.0 & 0.3	4	78,386		

^# ^Post test examination revealed a fracture through the second hole, superior to the lag screw

^+^Lateral condyle bone segment shifted laterally until purchase on the distal locking screw became sufficient to bend it

#### Biomet Matthews Nails

Of the two nail specimens tested at 2.0 kN (max. of cycle) specimen, 1 was a run out (end of test reached without failure), whilst specimen 2 failed after 315,043 cycles. The failure resulted from the lateral locking screw fracturing at the distal point of entry into the nail.

**Figure 7:**
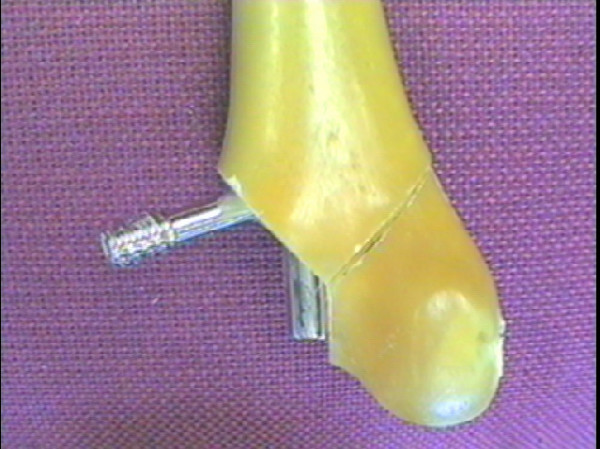
Photograph demonstrating a bent lateral locking screw with nail (Specimen 3).

**Figure 8:**
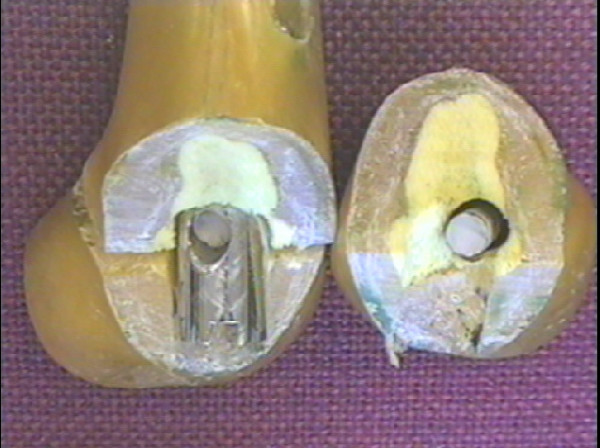
Photograph demonstrating a fractured distal locking screw with nail (Specimens 4).

Two specimens were tested at 3.0 kN with both failing in different ways; the failure of one specimen at 22,356 cycles resulted from the lateral condyle bone segment shifting laterally until the purchase on the distal locking screw became sufficient to bend it (Figure 7). This was due to improper reduction. Failure of the second specimen at 78,386 cycles resulted from the medial locking screw fracturing at the distal point of entry into the nail (Figure 8).

#### Dynamic Compression Screw

**Figure 9:**
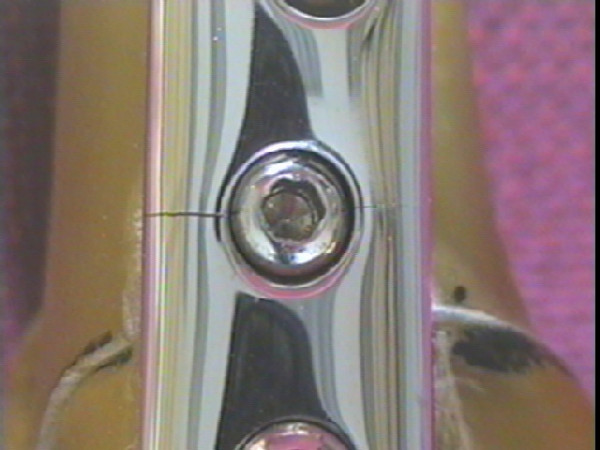
Photograph demonstrating a fracture through a screw hole with a screw in-situ.

**Figure 10:**
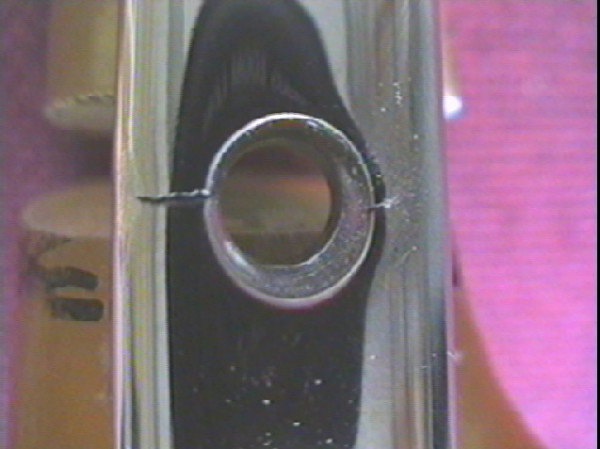
Photograph demonstrating a fracture through a screw hole with the screw removed.

Two specimens were tested at 2.0 kN with failure occurring in both, through the 2nd plate hole (Figures 9 &10). The failure was noted at the end of the test at 1 M cycles. Clearly therefore the endurance limit load for plates tested in this way is less than 2.0 kN.

### Endurance testing of the Biomet Matthews Nail and the Dynamic Compression Screw with transverse osteotomised composite femurs (Table 2)

**Table 2:** Endurance testing of the Biomet Matthews Nail and the Dynamic Compression Screw tested with transverse osteotomised composite femurs.

	**Biomet Matthews Nail with transverse osteotomised femur**		**Dynamic Compression Screw with transverse ostetotomised femur**	
**Max. & Min. of Load Cycle (kN)**	**Specimen Number**	**Cycles Completed**	**Specimen Number**	**Cycles Completed**

1.50 & 0.15	-	-	1	26,717
1.50 & 0.15	-	-	2	357,522
2.0 & 0.2	1	1,000,000		
3.0 & 0.3	2	1,000,000		
4.0 & 0.4	3	~14,500*		
4.0 & 0.4	4	~21,000*		

* Test aborted due to deterioration of femoral head in loading socket

#### Biomet Matthews Nail

Neither of the two specimens tested at 2.0 and 3.0 kN (max. of cycle) failed. This was also the case at 4.0 kN, but both tests at this load level had to be aborted at approximately 14,000 cycles and 21,000 cycles, due to the deterioration of the femoral head in the loading socket.

#### Dynamic Compression Screw

Two specimens were tested at 1.5 kN, with failure occurring in both at close to 30,000 cycles. This occured through the plate hole at the level of the osteotomy.

## Discussion & Conclusion

The insertion of an intramedullary nail is seen as a less technically demanding procedure than a dynamic compression screw [10]. It includes a shorter operative and soft tissue retraction time, less soft tissue dissection and periosteal stripping and reduced blood loss [10]. By protecting the soft tissues and periosteum the blood supply to the bone is preserved and healing is improved. This significant reduction in dissection may allow earlier mobilisation and rehabilitation of the patient. Until recently the considerations in managing distal femoral fractures was seen as a trade of between these highlighted benefits of the intramedullary nail and the superior rigid stability conferred by the dynamic compression screw [10].

In an early clinical study using supracondylar intramedullary nails in distal femoral fractures Innacone et al [11] was cautiously enthusiastic about their surgical experience, but suggested that additional biomechanical testing needed to be undertaken before widespread use.

A study using composite femurs by Firoozbakhsh et al demonstrated superior rigid stability with the DCS, yet the intramedullary nail performed well enough to be considered as a reasonable biomechanical alternative in clinical practice. Since then many new designs of retrograde intramedullary nails have been used in clinical practice and it is now a well recognised procedure to treat distal femoral fractures [7,10,12-14]. In our study the results with the transverse osteotomised femurs indicate that the Biomet Matthews Nail has an endurance limit load of at least twice that of the DCS under the test conditions.

A limitation of our study was the number of modes of loading used. In Firoozbakhsh's study the devices were tested in six different modes of loading including compression in varus and valgus, axial torsion, medial and lateral bending and bending in flexion. We also tested the devices using composite femurs but not on human cadaveric bone.

Zlowodzki et al [15] and Meyer et al [16] used a similar design of intramedullary nail to Firoozbakhsh et al, in cadaveric bone and confirmed their finding of reduced rigid stability in axial and torsional loading.

These previous studies looked at the biomechanical stability of the supracondylar (extra-articular) fractures. However the most challenging fracture for the surgeon to treat is the Y shaped bicondylar (intra-articular) fracture [1]. In the Y fractured femurs both Dynamic Compression Screws tested at 2.0 kN failed, whilst of the two Biomet Matthews Nails tested at this load, one failed and one did not. This therefore suggests that the Biomet Matthews Nail has an endurance limit load which is as great, or greater than that of the dynamic compression screw under the test conditions. It is this fracture configuration that the new Biomet Matthews Nail was specifically designed to treat. The unique "cruciate" orientation of the distal locking screws allow them to pass through the Biomet Matthews Nail and gain the maximum bone purchase in the femoral condyle and therefore improving the stability of the fracture fixation. As these fractures are usually in weak osteoporotic bone, this *in vitro *study indicates that the Biomet Matthews Nail is a favourable surgical option, in these patients. The Nail has been given an EC Declaration of Conformity and the clinical results of the first fifty nails are currently being submitted for publication.

## Competing interests

MGM holds the patent for this device and is working in conjunction with Biomet in its production. PS works for Biomet Ltd UK as an engineer.

## Authors' contributions

LOR, JD & BD reviewed the results, published literature and co-wrote article. MGM designed the Biomet Matthews Nail and implanted the nails and dynamic compression screws. PS performed the mechanical testing of the devices and co-wrote the article. All authors have read and approved the final manuscript.
